# Supplementary material for: Acetylcholine Sustains LNCaP Prostate Cancer Cell Migration, Invasion and Proliferation Through Glyoxalase 1/MG-H1 Axis with the Involvement of Osteopontin
Source: Int J Mol Sci. 2025 Apr 25;26(9):4107. doi: 10.3390/ijms26094107 (PMC12072008; doi:10.3390/ijms26094107)
Supplement: Supplementary file 1 [file ijms-26-04107-s001.zip › ijms-3519306-supplementary.pdf]

**Supplementary Material: Acetylcholine sustains LNCaP prostate cancer cell migration, invasion and proliferation through glyoxalase 1/MG-H1 axis with the involvement of osteopontin**

**Dominga Manfredelli [1], Tatiana Armeni [2], Lidia De Bari [3], Andrea Scirè [4], Vincenzo Nicola Talesa [1], Cinzia Antognelli [1,*] and Marilena Pariano [1]**

**Figure S1.** Acetylcholine (ACh) levels in LNCaP cells upon treatment with exogenous ACh, Donepezil (DNPZ) and a combination of both. ACh was measured using a specific ELISA kit as described in Materials and Methods section. LNCaP cells were pre-treated with 5 μM DNPZ for 24 hours and then exposed to 5 μM Acetylcholine (ACh) for additional 24 hours. The histogram represents the mean ± SD of three independent experiments. ****$p < 0.0001$.

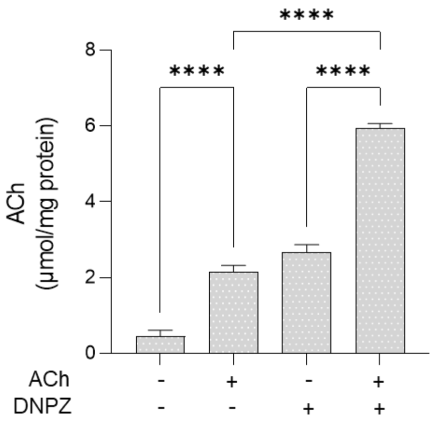


**Figure S2.** Whole gel for panel b of Figure 7

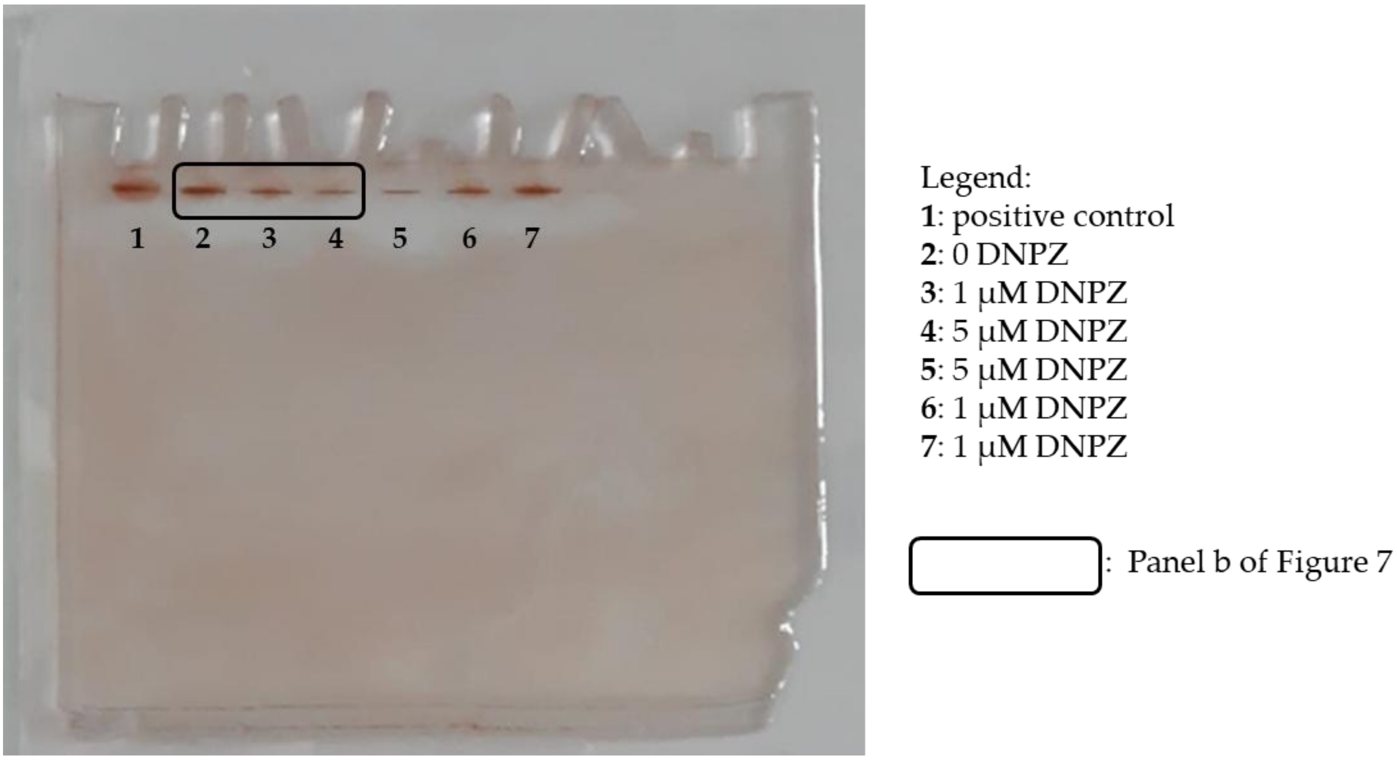

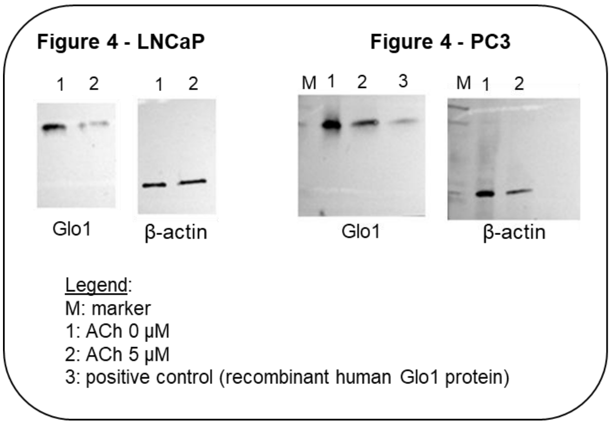


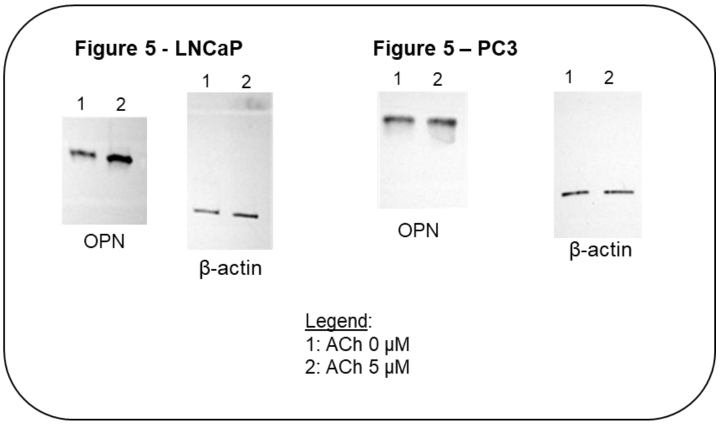


**Figure 4 and Figure 5 - Original blots**
